# Back-table specimen scanning using gantry-free hybrid *h*SPECT/LiDAR imaging: a feasibility study during PSMA-radioguided surgery

**DOI:** 10.1007/s00464-025-12081-w

**Published:** 2025-08-25

**Authors:** Giusi Pisano, Matthias N. van Oosterom, Vera A. Ottens, Anne-Claire Berrens, Leon J. Slof, Berkay A. Çakal, Daphne D. D. Rietbergen, Henk G. van der Poel, Pim J. van Leeuwen, Fijs W. B. van Leeuwen

**Affiliations:** 1 Interventional Molecular Imaging Laboratory, Leiden University Medical Centre, Leiden, The Netherlands; 2 Nuclear Medicine Unit, Fondazione Policlinico Universitario A. Gemelli IRCCS, Rome, Italy; 3 Department of Urology, Netherlands Cancer Institute-Antoni Van Leeuwenhoek Hospital, Amsterdam, The Netherlands; 4 Design and Prototyping, Department of Medical Technology, Leiden University Medical Centre, Leiden, The Netherlands; 5 Clinical Computational Medical Imaging Research, Department of Diagnostic and Interventional Radiology and Neuroradiology, University Hospital Augsburg, Augsburg, Germany; 6 Crystal Photonics GmbH, Berlin, Germany; 7 Section of Nuclear Medicine, Department of Radiology, Leiden University Medical Center, Leiden, the Netherlands; 8 Department of Urology, Amsterdam University Medical Center, VUmc, Amsterdam, The Netherlands

**Keywords:** Radioguided surgery, Prostate cancer, Specimen scanning, PSMA SPECT/CT/LiDAR, Surface scanning, Image-guided surgery

## Abstract

**Introduction:**

Prostate-specific membrane antigen (PSMA) targeted precision surgery is becoming increasingly popular. However, the relatively low levels of PSMA-receptor expression and background signal can hinder in vivo lesion detection and margin evaluation. Back-table imaging (ex vivo) potentially provides a means to confirm surgical accuracy. For ^99m^Tc-PSMA-radioguided surgery, an innovative gantry-free hybrid imaging technique has recently been proposed, namely handheld single-photon emission computed tomography (*h*SPECT) combined with light detection and ranging (LiDAR). This study aimed to assess the feasibility and performance of *h*SPECT/LiDAR in analyzing tissue specimens excised after robotic ^99m^Tc-PSMA-radioguided surgery.

**Methods:**

We included samples from 5 prostate cancer patients undergoing primary or salvage robot-assisted resection of ^99m^Tc-PSMA-I&S avid lesions that were identified using a drop-in gamma probe. 12 samples (1 prostatic tissue, 1 local recurrence tissue, 10 lymph nodes) were analyzed ex vivo using a custom-built specimen tray, including an optical reference tracker for scan registration. LiDAR was used to acquire a surface scan of the specimens, and the 3D OBJ image output was fused with the 3D DICOM of a *h*SPECT obtained using a handheld gamma camera and DeclipseSPECT tracking system.

**Results:**

*h*SPECT/LiDAR imaging provided accurate representation of the ^99m^Tc-PSMA-I&S uptake within the specimens. In 8 samples, it helped to confirm a true positive lesion. In the remaining 4 samples, non-visualization aligned with negative histopathology (true negative). A strong correlation was found between PSMA-*h*SPECT/LiDAR and PSMA-PET/CT (*p* < 0.05), but no correlation could be established with PSMA-SPECT/CT (*p* = 0.515). The count rates fount in the scan correlated to tumor size (*p* = 0.016) and were not influenced by the overall specimen’s size (*p* = 0.558).

**Conclusion:**

We present the technical feasibility of a new 3D hybrid modality (*h*SPECT/LiDAR) that allows back-table assessment of surgical specimens from the already well validated robotic ^99m^Tc-PSMA-radioguided surgery workflow.

**Supplementary Information:**

The online version contains supplementary material available at 10.1007/s00464-025-12081-w.

Prostate-specific membrane antigen (PSMA) is a transmembrane glycoprotein that is highly overexpressed on the surface of prostate cancer cells. Unlike the prostate-specific antigen (PSA) serum biomarker, PSMA is not detected in the bloodstream but serves as an ideal target for molecular imaging, radioguided interventions and therapy [1]. PSMA-based diagnostics are dominated by the use of positron emission tomography (PET), a nuclear imaging application that has been able to surpass conventional radiological imaging modalities (i.e., Magnetic resonance imaging–MRI or Computed tomography–CT) [2–4]. To guide surgical resection of patients that display PSMA-positive local disease, various gamma-ray emitting PSMA-analogues have been developed, examples being ^111^In-PSMA-imaging and therapy (I&T) and ^99m^Tc-PSMA-imaging and surgery (I&S) [5–7]. The wide availability of surgical radioguidance modalities that are compatible to these low-to-mid energy gamma-emitting radioisotopes has meant that these radiopharmaceuticals dominate global PSMA-targeted surgery efforts [8, 9]. In particular ^99m^Tc-PSMA-I&S has shown promise during primary [10, 11] and salvage [12, 13] surgery, having seen implementation in more than 553 patients and at least 4 countries [8, 14]. In these procedures, lesion detection is facilitated by either a conventional gamma probe (open surgery) or a dedicated drop-in gamma probe for the increasingly popular robotic resections [15–17].

As PSMA-biology underlies the uptake of PSMA-targeting radiopharmaceuticals, the degree of receptor expression on tumor cells dictates the accumulation of said radiopharmaceuticals. Such receptor targeted strategies can lead to relatively low signal intensities, that influence the intraoperative detection [10, 11, 17], especially in case of small metastases and during margin assessments. The pharmacological clearance of ^99m^TcPSMA-I&S can complicate detection even further, with background signal from urinary tract and intestines that overlaps with the pelvic surgical field [18, 19]. As targets are separated from background signals and tend to be more accessible for detectors when fully excised, ex vivo examinations on a back-table in the surgical room is generally used to confirm, or sometimes even replace intraoperative analysis.

To guide pathological margin assessments, small-bore PET-gantries have been used to visualize beta-emitting radiopharmaceuticals such as ^68^Ga-PSMA-11 and ^18^F-PSMA-1007 [20–22]. Ideally back table tissue examinations align with in vivo image guidance technologies. Unfortunately, intraoperative use of high-energy beta-emitting radiopharmaceuticals can increase the staff exposure to ionizing radiation [23]. Therefore, it would make sense to pursue the ex vivo tissue examination of the common PSMA-radioguided resections that tend to rely on ^99m^Tc-PSMA radiopharmaceuticals (see above) [8, 9].

We hypothesized that the Declipse single-photon emission computed tomography - *handheld*SPECT (hSPECT) [24], a CE-marked and clinically proven augmented and virtual reality platform designed for radioguided surgery with gamma-emitting radiopharmaceuticals, can also support specimen scanning by combining it with handheld light detection and ranging (LiDAR). This combination supports a novel hybrid modality which helps to display the radioactive volume within the surface-contours of the excised specimens. Following an initial case report [25], we have now extended our evaluation of operational feasibility.

## Methods

### Patients

A feasibility study was set-up to evaluate the technical performance of the hybrid *h*SPECT/LiDAR imaging modality; therefore, no randomization was performed and the CONSORT reporting criteria do not apply. The Netherlands Cancer Institute-Antoni van Leeuwenhoek (NKI-AvL) institutional review board approved this study (IRBdm24-249). The samples for this study were included from 5 prostate cancer patients who showed positive lesions on PSMA-PET/CT and were selected for primary or salvage robot-assisted ^99m^Tc-PSMA-I&S-guided surgery between April 2024 and December 2024. Adult patients were included for primary surgery if they had pathologically confirmed, non-distant-metastatic (M0) prostate cancer, non-eligible for active surveillance according to EAU guidelines [26]. Patients underwent salvage surgery if they had hormone-sensitive recurrent prostate cancer after robot-assisted laparoscopic prostatectomy (RALP) or primary radiotherapy (brachytherapy or external beam radiotherapy) with or without pelvic lymph node dissection (PLND), with involvement of ≤ 2 lymph nodes (LNs) or local oligorecurrent disease in the pelvis at PSMA-PET/CT [27].

### Clinical workflow

**Fig. 1:**
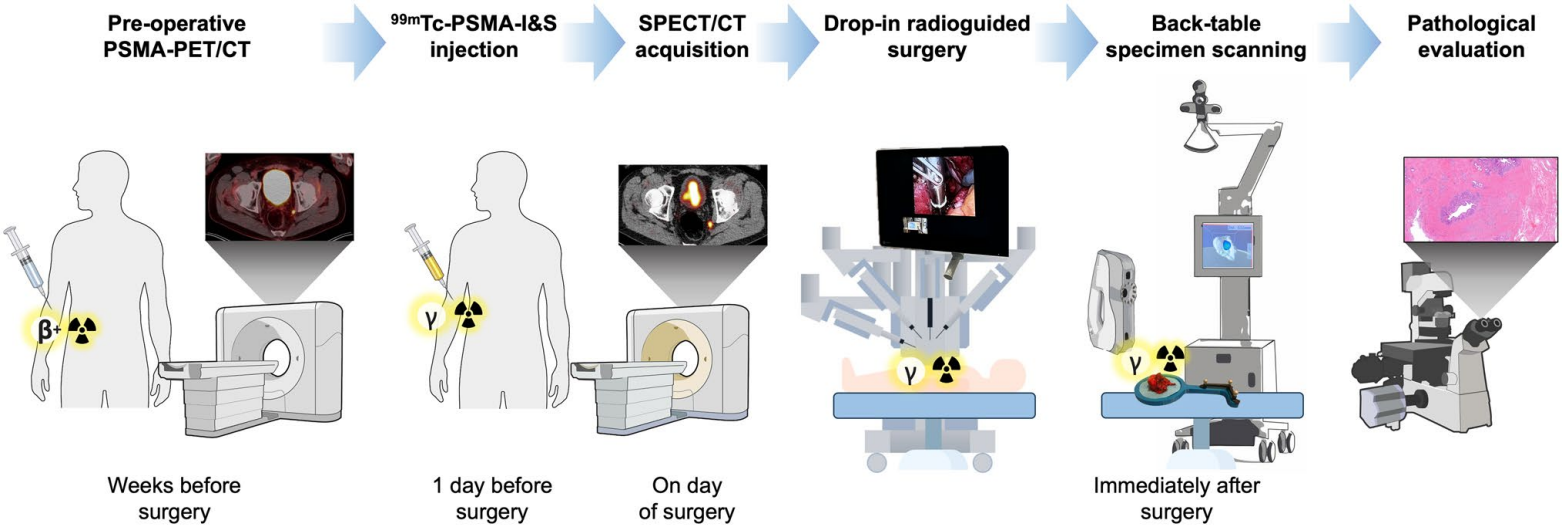
Clinical workflow of this study starts with patients undergoing a diagnostic PSMA-PET/CT weeks before the procedure, for staging or follow-up purpose. If selected for robot-assisted radioguided surgery, patients underwent ^99m^Tc-PSMA-I&S intravenous injection the day before surgery, and SPECT/CT acquisition on the morning of surgery. Intraoperatively, radioactivity was detected through the drop-in probe during robot-assisted surgery. After removal of the specimens, the ex vivo counts were measured using a handheld gamma probe and subsequently examined by a specimen scan before being sent to pathology for final analysis

The clinical workflow for patients (see Fig. 1) began with diagnostic PSMA-PET/CT for staging purposes (^18^F-JK-PSMA-7 or ^18^F-PSMA-7), performed 1–3 months before surgery (average 70 days before surgery, range 41–89 days). In patients that were included in the study, ^99m^Tc-PSMA-I&S was administered intravenously the day prior to surgery (mean injected activity of 565.81 MBq; SD 32.6; range 531–599 MBq). On the morning of the surgery, a preoperative ^99m^Tc-PSMA SPECT/CT was acquired. The patients then underwent robot-assisted radioguided surgery using the Da Vinci Xi® robotic system (Intuitive Surgical®, Sunnyvale, United States) and a drop-in gamma probe (Crystal Photonics GmbH, Berlin, Germany). Once resected, ex vivo counts were measured using a handheld gamma probe (Neoprobe®, Navidea Biopharmaceuticals, Dublin, OH, USA). Subsequently, without additional tissue preparation, ex vivo specimen scanning was conducted before the samples were sent for pathological evaluation (Fig. 1).

### Specimen scanning

**Fig. 2:**
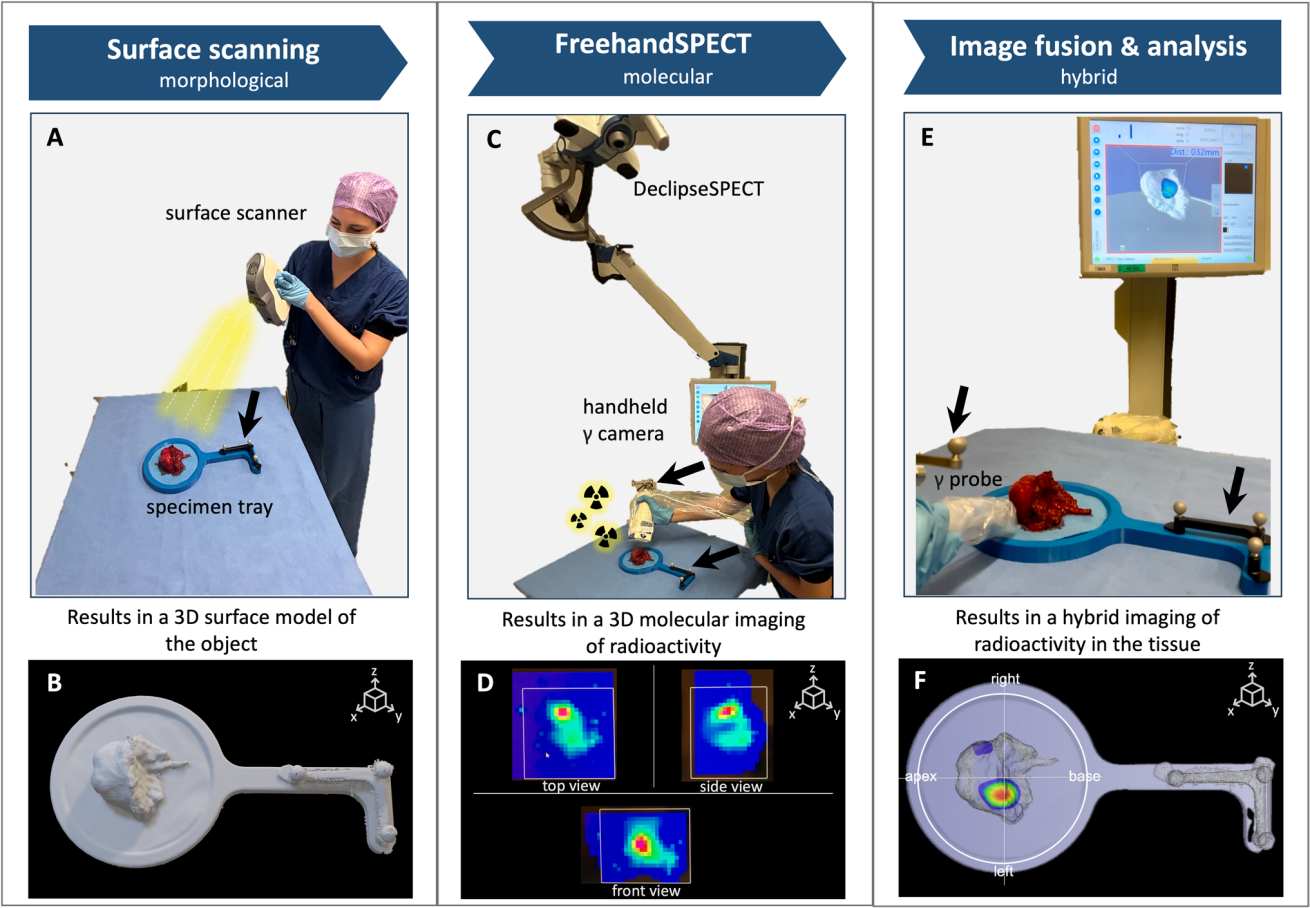
The setup and instrumentations of the specimen scanning are depicted in sequence, in the case of a primary prostate specimen. First of all, surface scanning of the tissue (Artec Eva) was performed (black arrows indicate optical reference tracker) (**A**) to obtain a 3D surface model of the excised tissue specimen (**B**). Gantry-free SPECT was acquired using a declipseSPECT system combined with an optically tracked CrystalCam (**C**) to generate the images of ^99m^Tc-PSMA distribution (**D**). Lastly, the LiDAR scan and the molecular ^99m^Tc-PSMA *h*SPECT were fused and visualized, enabling a real-time examination of the tissue from different angles employing a handheld gamma probe as pointer (**E**). As a final result, a hybrid imaging of the radioactivity distribution within the tissue surface was generated (**F**)

Specimens were placed on a custom-built specimen tray, with a slot for tissues and integrated optical reference tracker for registration of both scans. Thus, allowing single-object scanning and focused field-of-view. To provide reference of the sample surface, a tissue surface scan was created using a handheld Artec Eva® LiDAR scanner (Artec3D®, Luxembourg; 0.5 mm resolution; dimensions: 262 × 158 × 64 mm) [28, 29]. To visualize the tissue avidity for ^99m^Tc-PSMA-I&S, a molecular freehand SPECT was generated using the mobile declipse®SPECT (SurgicEye® GmbH, Berlin, Germany; dimensions: ~ 60 × 100 × 165 cm when folded for transportation; ~ 60 × 150 × 200 cm when unfolded) system in combination with an optically tracked handheld CrystalCam (Crystal Photonics GmbH; dimensions: 65 × 65x180 mm) [24, 30]. A 3-mm pixel spacing with isotropic voxels was employed, using an iterative reconstruction algorithm (maximum-likelihood expectation maximization, 20 iterations). The LiDAR scan and handheld ^99m^Tc-PSMA SPECT took approximately 3–4 min each, for a total of 6–8 min for the complete scan for each specimen. The surface Object file (OBJ) output of the handheld LiDAR was combined with the 3D DICOM of the SPECT, and the registration was realized using the asymmetrical refence trackers. Visualization and analysis of the hybrid images occurred in 3D Slicer software (version 5.6.2, http://www.slicer.org [31]), that also allowed automatic fusion of the images and to adjust the signal threshold levels. The fused 3D images then helped support augmented or virtual reality displays, wherein an optically tracked handheld gamma-probe (Crystal Photonics GmbH) could be used as pointer, allowing investigation of the tissue from different angles, providing distance estimates between the probe tip and the radioactive signal in the video-view (Fig. 2).

### Pathological analysis

Immediately after back-table specimen scanning, the surgical samples were sent for pathological analysis with hematoxylin and eosin (H&E) staining as per standard clinical protocol. Pathology was considered as the gold standard to define true- and false-positives (TP, FP), as well as true- and false-negatives (TN, FN).

### Statistical analysis

Descriptive statistics were reported in terms of mean, standard deviation (SD), range or proportion. To assess the association between pre-operative imaging findings and back-table specimen scanning, data were organized into a 2 × 2 contingency table. Given the small sample size, Fisher’s Exact Test was employed to determine whether there was a statistically significant association between the variables. To assess the relationship between specimen size (measured as the major axis in millimeters) vs ex vivo counts per second (cps) and metastases size (in millimeters) vs ex vivo cps, Spearman's rank correlation coefficient was used. The strength and significance of the correlation were evaluated using the correlation coefficient (*ρ*) and corresponding p-value. Statistical significance was set at *p* < 0.05. The positive predictive value (PPV) was calculated as TP / (TP + FP). All statistical analyses were performed using R software, version 4.4.2.

## Results

In total, 5 patients who underwent ^99m^TcPSMA-I&S radioguided surgery were included. One patient was included for primary surgery of a prostate adenocarcinoma found at pre-operative biopsy (Gleason Score (GS) 4 + 3 = 7). In this patient, 2 PSMA-avid lesions were resected (the primary tumor and one lymph node). The other 4 patients underwent salvage surgery for disease recurrence, where on average 1.5 PSMA-avid lesions were resected per case. Real-time decision-making during lesion resection was based on radioguided surgery, with findings subsequently confirmed with handheld (*h*) *h*SPECT/LiDAR. In total from the 5 cases, 12 samples were investigated (1 prostatic tissue, 1 local recurrence tissue, 10 lymph nodes) with *h*SPECT/LiDAR, including both the PSMA-avid and non PSMA-avid-lesions as controls (see Table 1). Indeed, *h*SPECT/LiDAR was employed to confirm the absence of radioactivity in control lesions and to verify the presence and distribution of signal in suspicious lesions.

**Table 1:** Patients’ and specimens’ characteristics

Case n	Age at surgery	Type of surgery	Clinical data	Injected activity ^99m^Tc-PSMA-I&S (MBq)	Specimen n	Specimen type	Specimen size (major axis, mm)	Ex vivo cps	PSMA-PET/CT	PSMA-SPECT/CT	hSPECT/LiDAR	Pathology
1	71	Primary (RALP + ePLND)	-Preop. biopsy GS 4 + 3 = 7 cT2aN1M0	531	1	Prostate	68.18	Nr	+	+	+	Tumor positive (GS 4 + 5 = 9)
					2	External iliac LN	15.08	13	-	-	-	Tumor negative
					3	Obturator LN	21.38	300	+	+	+	Tumor positive (9.2 mm)
2	78	Salvage	-2012: RALP pT3bNxMx GS 4 + 3 = 7 -2013: biochemical recurrence (external RT)	591,5	4	Pararectal LN	56.00	67	+	-	+	Tumor positive (12.2 mm)
					5	External iliac LN	35.50	15	+	-	+	Tumor positive (5.6 mm)
3	70	Salvage	-2017: RALP + PLND, pT2aN0Mx R0 GS 3 + 4 = 7 -no additional therapy	541,74	6	Local recurrence (Soft tissues)	40.28	141	+	-	+	Tumor positive
					7	External iliac LN	45.85	92	+	-	+	Tumor positive (4 mm)
					8	External iliac distal LN	33.26	9	-	-	-	Tumor negative
4	65	Salvage	-2022: RALP + PLND pT3aN0M0 R0 GS 4 + 4 = 8 -2023: start ADT	599	9	Internal iliac LN	23.03	16	-	-	-	Tumor negative
					10	Pararectal LN	42.33	140	+	-	+	Tumor positive (7 mm)
5	71	Salvage	2012: RALP + LN dissection PT2aN1Mx GS 3 + 4 = 7 -no additional therapy	595.29	11	Internal iliac LN	21.96	135	+	-	+	Tumor positive (5.7 mm)
					12	Cloquet’s LN	14.05	21	-	-	-	Tumor negative

*LN* Lymph node, *RALP* Robot-assisted laparoscopic prostatectomy, *(e)PLND* (extended) Pelvic lymph node dissection, *ADT* Androgen deprivation therapy, *RT* Radiotherapy, *GS* Gleason score, *Nr* not reported

**Fig. 3:**
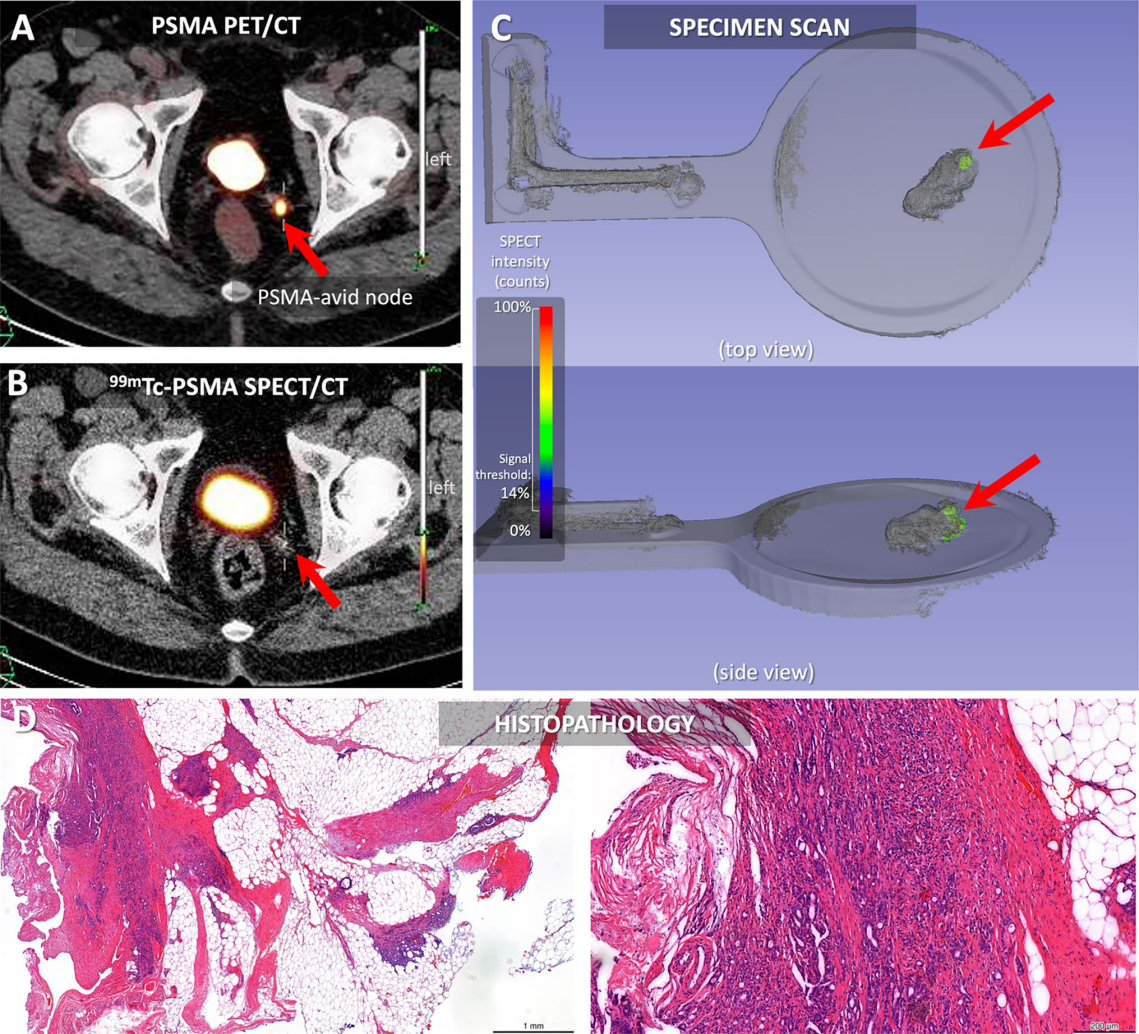
Case 3 from Table 1–Displaying a case of recurrent cancer, of a patient who underwent RALP and pelvic lymph node dissection in 2017 for a GS 7 (3 + 4) acinar adenocarcinoma with no additional therapies. At that time, pathology resulted in a pT2aN0Mx, R0. At follow-up PSMA-PET/CT imaging, a PSMA-avid node was seen in the left pelvis (**A**, red arrow), not intense on SPECT/CT (**B**). At *h*SPECT/LiDAR specimen scanning, radiotracer uptake was visible inside the tissue (**C**, red arrow). At histopathological analysis (**D**, H&E left 1 mm magnification, right 200 μm magnification), the specimen showed to contain thick nerve bundles surrounded by acinar type adenocarcinoma, with adenocarcinoma localized also in the fibrous tissue. Again, the positive histology correlated with our images

All the specimens (prostate specimen size: 68.18 mm; other specimen mean size 31.7 mm, SD 13.6; range 14.05–56 mm) could be imaged in the surgical facility using *h*SPECT/LiDAR imaging. All 8 specimens found to be positive at pathology (metastases size ranging from 4 to 12.2 mm), could be resected under radioguidance and had their ^99m^Tc-PSMA-I&S accumulation successfully be visualized using *h*SPECT/LiDAR (see Table 1 and Fig. 3).

Overall, a total of 8 TP and 4 TN were found a specimen scanning, with no FP or FN findings, and a PPV of 100% (see Table 1). The 8 positive specimens were positive at PET/CT, showing a strong correlation between PET/CT imaging and *h*SPECT/LiDAR (Fisher’s Exact Test p-value < 0.05). Conversely, pre-operative SPECT/CT could only clearly identify 2 (25%) out of the 8 PSMA-PET/CT positive samples. Thus, indicating back-table *h*SPECT/LiDAR of specimens yielded a superior sensitivity over preoperative SPECT/CT and no significant correlation was found between the two modalities (Fisher’s Exact Test *p*-value = 0.515).

During investigation of the *h*SPECT/LiDAR scans, the minimum radioactive signal threshold was set at 10–16%, to allow for a successful correlation between the specimen scanning with histopathology, pre- and intra-operative imaging. Whereby the primary tumor, margins were correctly assessed as tumor free. The 4 negative samples at specimen scanning were also negative at PSMA-PET/CT and PSMA-SPECT/CT and were confirmed as non-metastatic at pathology.

**Fig. 4:**
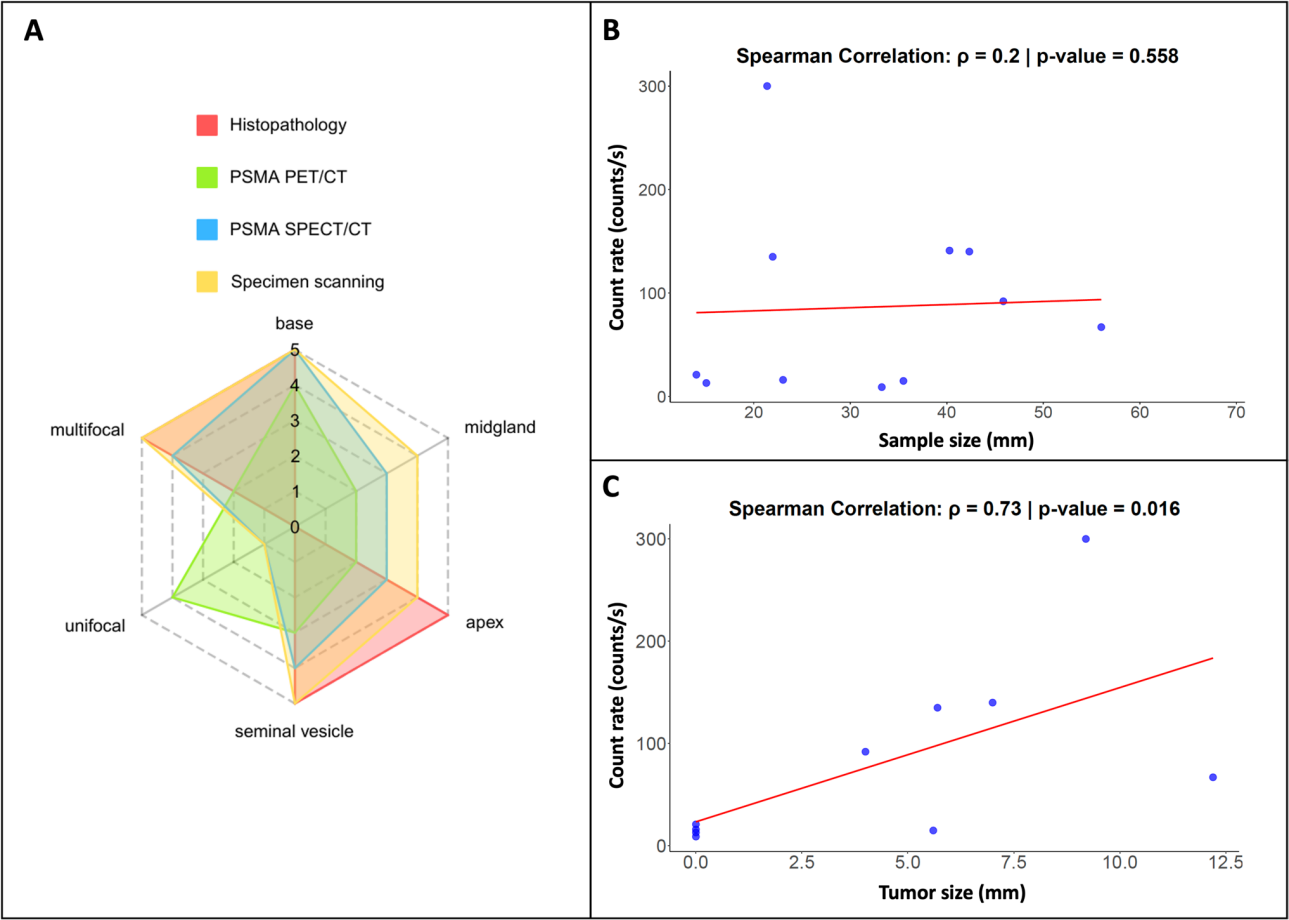
**A** Radar chart of the primary prostate cancer case (Case 1 from Table 1) depicting radioactivity localization by *h*SPECT/LiDAR specimen scanning, PSMA-PET/CT, PSMA-SPECT/CT. Each axis represents a different variable: prostate zones (base, midgland, apex), focality (unifocal vs. multifocal), and involvement of seminal vesicles. The radial scale (from 0 to 5) represents the score of radioactivity localization for the modalities. A score of 0 suggests no radioactivity (negative), and a score of 5 indicates strong radioactivity (positive). The same parameters were correlated with tumor presence at histopathology (0: negative, 5: positive). **B** and **C** Scatter plot illustrating the relationship between respective specimen size (mm, x-axis) or tumor size (mm, x-axis) with count rate (counts/s, y-axis). Each data point represents an individual observation. A linear regression trend line (red) has been applied to visualize the overall pattern

A significant positive correlation was found between the count rates and the size of metastases (Spearman correlation coefficient *ρ* = 0.732, *p*-value = 0.016, Fig. 4). As such the ex vivo ^99m^Tc-PSMA-I&S count rates indicate that the biological expression of PSMA is associated with the tumor volume. Further data analysis also revealed that the count rates (range 9 to 300 counts/s) were not dependent on the specimens’ size (Spearman correlation coefficient *ρ* = 0.2; *p*-value = 0.558, Fig. 4), indicating signal attenuation was limited. At follow-up, no patient showed recurrence (Supplementary Table 1).

When we investigated the spatial correlation of the different diagnostic findings for case 1 (Fig. 4), we found a higher score particularly at the prostate base for all the 3 imaging modalities, suggesting the presence of tumor in that zone, which was confirmed at pathology. PET/CT seemed to indicate unifocal involvement, while both SPECT/CT and *h*SPECT/LiDAR yielded two areas of involvement, in line with the bilateral adenocarcinoma confirmed at final analysis. Moreover, *h*SPECT/LiDAR accurately mapped infiltration by adenocarcinoma in proximity to the seminal vesicle, a feature that was less prominent on PET/CT and SPECT/CT. Combined, specimen scanning resulted in stronger positivity scores, confirmed at pathology, for base, apex, multifocality and seminal vesicles tumor involvement (Fig. 4).

**Fig. 5:**
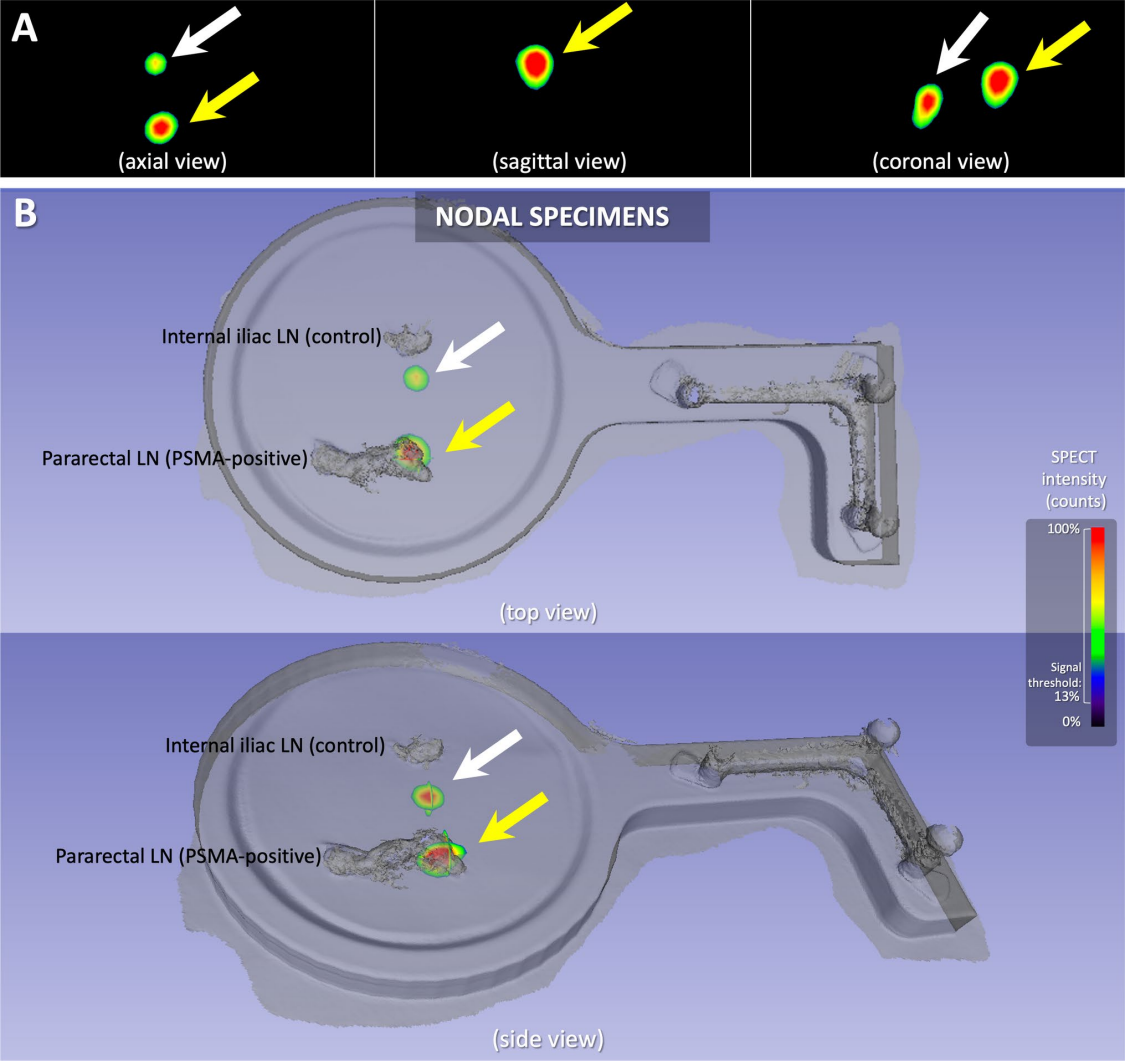
Case 4 from Table 1- In this recurrence case, two nodes were scanned with *h*SPECT/LiDAR after robot-assisted radioguided surgery, one pararectal LN (suspicious) and one internal iliac LN (control). At 3D Slicer visualization of radioactivity **A**, two spots of signal could be seen at axial and coronal views (white and yellow arrows), that could have been attributed to the two LNs. At specimen scanning hybrid display **B**, the highest radioactivity spot clearly felt into the pararectal LN (yellow arrow), while the other spot being possibly attributable to a contamination on the specimen tray (white arrow)

The value of *h*SPECT/LiDAR is further underscored by the case presented in Fig. 5 (Case 4 from Table 1). Here, the surface scan helped to discriminate signal in the node form, most likely, a contamination on the specimen tray. Without the surface context of the tissue samples, both nodes would have been assumed to be positive. At histopathology, the pararectal LN displayed adenocarcinoma metastatic involvement (7 mm) (TP), while the internal iliac LN was confirmed as indeed tumor negative (TN).

## Discussion

Our findings demonstrate that ^99m^Tc-PSMA-specimen scanning using *h*SPECT/LiDAR imaging is technically feasible and compatible with the surgical workflow. The findings also correlate with routinely applied analysis such as: H&E histopathology, ^99m^Tc-PSMA-I&S radioguided surgery, and PSMA-PET/CT.

When evaluating concordance, all the positive lesions at *h*SPECT/LiDAR specimen scans were also positive at PET/CT and histology (8 TP), while the negative ones were negative at PET/CT and histology (4 TN). Instead, the concordance between PSMA-PET/CT and PSMA-SPECT/CT as well as between PSMA-SPECT/CT and PSMA-*h*SPECT/LiDAR were poor. The superior SPECT sensitivity that can be obtained in the surgical theatre appears to be caused by the ability to place a 4 × 4 cm^2^ CrystalCam detector in a 2–3 cm vicinity of already isolated targets. In comparison, a ~ 40 cm × 50 cm^2^ SPECT/CT detector needs to detect lesions at approximately 15–30 cm distance and has to do so in the facility of background signals (in e.g., clearance organs) [32–35]. Moreover, when tissues are scanned ex vivo, they are spatially more accessible and the effect of tissue-attenuation is minimal [15, 36]. With that, *h*SPECT/LiDAR better aligns with PSMA-PET/CT, the standard for PSMA-diagnostics (∼3–5 mm resolution) and pooled sensitivity of 0.97 for ^68^Ga-PSMA) [37–40].

Literature indicates that findings in surgical margins and nodal metastases obtained with small-bore PET/CT gantries align with pre-operative PSMA-PET/CT imaging and histopathology. Darr et al. employed signal from ^68^Ga-PSMA-11 (172 MBq injection 5.2 h prior to specimen scan) or ^18^F-PSMA-1007 (223.5 MBq injection 6.5 h prior to specimen scan). They found that 93% of lesions detected at PSMA-PET/CT were also positive at specimen PET/CT, resulting in a significant correlation (Pearson coefficient of 0.935, *p*-value = 0.001) [20]. Moraitis et al. employed ^18^F-PSMA-1007 (3.7 MBq/kg 4.6 h prior to specimen scan) specimen PET/CT yielding a correlation coefficient of positive surgical margins with histopathology of 0.90 (p-value < 0.001) [22]. Our results with *h*SPECT/LiDAR showed 100% of lesions detected at PSMA-PET/CT were also positive at *h*SPECT/LiDAR, resulting in a significant correlation (*p*-value < 0.05). Moreover, the absence of false-positive findings meant that all tumor-positive results were true positives, yielding a 100% PPV.

What is markedly different in the reports that describe use of small-bore PET/CT gantries, is that they present cases whereby the resections of target tissues were not aided by radioguidance. This, despite the fact that beta-radioguidance benefits from a direct alignment with PET/CT and matching radiochemical designs [23] and has proven to be clinically feasible, even in a robotic setting [41]. In our case radioguided surgery, an already widely implemented concept [8], defined the surgical resection, providing an internal reference for *h*SPECT/LiDAR. The significant positive correlation observed between ex vivo ^99m^Tc-PSMA-I&S count rates and metastatic lesion size (Spearman’s *ρ* = 0.732, *p*-value = 0.016) highlights how radiotracer uptake corresponds closely with tumor burden and biological PSMA expression.

Conversely, the *h*SPECT/LiDAR approach was used to complement an already routine ^99m^Tc-PSMA-I&S radioguidance procedure [8, 9, 23], and can equally be implemented with other gamma-emitting radiopharmaceuticals (for e.g., the sentinel node tracer ICG-^99m^Tc-nanocolloid). The value in using ^99m^Tc comes from the superior tissue penetration (> 10 cm for 140 keV photons vs. ≤ 2 mm for positrons) and the negligible radiation exposure for the surgical staff [23, 42–44]. Because of that, the presented *h*SPECT/LiDAR modality can complement ongoing surgical paradigms using well-established and widely available surgical detection modalities, such as drop-in gamma probes [45], handheld gamma cameras (e.g., CrystalCam) [46, 47], DeclipseSPECT [24], and more recently gantry-free intraabdominal robot-assisted SPECT (_Robotic_SPECT) [48].

The integration of functional imaging modalities like PET or SPECT with morphological imaging (e.g., CT or MRI), has resulted in hybrid modalities that improve diagnostic accuracy [49]. Unfortunately, surgical rooms tend to be challenged for space. In that sense, our use of handheld LiDAR surface scanners align with trends seen in dentistry, maxillofacial surgery and orthopedics [50–52]. The mobile declipseSPECT platform, which can be moved between most operating environments and pathology, supports utility during intra- and post-operative imaging. Uniquely we have been able to show how *h*SPECT/LiDAR virtual reality displays on the Declipse platform can be used to provide crucial anatomical context for ^99m^Tc-PSMA-I&S uptake in surgical prostate cancer specimens (Fig. 5). From a cost perspective, the surface scanner is relatively affordable with lower acquisition and maintenance costs. Next to serving multiple uses, the declipseSPECT system is substantially cheaper than fixed SPECT/CT, PET/CT and micro-PET systems. These practical factors will favor technology adoption.

The limited cohort size and the lack of positive margins restrict the statistical power of correlating our back-table findings to outcome measures. Nevertheless, the alignment of the back table findings to PSMA-PET/CT, ^99m^Tc-PSMA-I&S radioguided surgery and H&E pathology indicates that the technology is capable of corroborating ^99m^Tc-PSMA-I&S distributions in excised tissue. This feature aligns with the general assumption that incomplete tumor resections are a routine cause for local recurrence [53, 54]. Moreover, the goal of our study was to evaluate technical capacity of *h*SPECT/LiDAR and its usability in a surgical complex. Therefore, the technique’s impact on intraoperative decision-making was not assessed. Such evaluations are part of future efforts that will require larger and more diverse patient bodies, thus enhancing the statistical power of outcome correlations, helping define optimal threshold levels, and helping determine how the technology impacts intraoperative decision-making.

## Conclusions

We presented a novel *h*SPECT/LiDAR hybrid imaging modality. A technology that seamlessly integrates in the well validated ^99m^Tc-PSMA-radioguided surgery workflow with a 100% PPV for back-table confirmation. Further studies are needed to investigate how specimen imaging impacts the surgical decision-making and oncological outcomes.

## Supplementary Information

Below is the link to the electronic supplementary material.

Supplementary file1 (DOCX 15 KB)
