# Supplementary material for: Back-table specimen scanning using gantry-free hybrid hSPECT/LiDAR imaging: a feasibility study during PSMA-radioguided surgery
Source: Surg Endosc. 2025 Aug 25;39(10):6943–54. doi: 10.1007/s00464-025-12081-w (PMC12500833; doi:10.1007/s00464-025-12081-w)
Supplement: Supplementary file 1 — Supplementary file1 (DOCX 15 KB) [file 464_2025_12081_MOESM1_ESM.docx]

**Supplementary Table 1:** Follow-up characteristics

| Case | Surgical margins | PSA nadir (ng/ml) | Total follow-up at NKI-AvL (months) | Number of PSA measurements | Last PSA measurement  (ng/ml) | Progression during follow-up at NKI-AvL |
| --- | --- | --- | --- | --- | --- | --- |
| 1 | R0 | 0.02 | 7* | 1* | 0.02* | No* |
| 2 | / | 0.01 | 12 | 2 | <0.01 | No |
| 3 | R0 | <0.01 | 11 | 3 | 0.01 | No |
| 4 | / | 0.07 | 8 | 2 | 0.08 | No |
| 5 | / | <0.01 | 7 | 1 | <0.01 | No |

*: Patient transferred to regional hospital
